# Oviposition of *Culicoides insignis* (Diptera: Ceratopogonidae) under laboratory conditions with notes on the developmental life history traits of its immature stages

**DOI:** 10.1186/s13071-021-05025-5

**Published:** 2021-10-09

**Authors:** Dinesh Erram, Nathan Burkett-Cadena

**Affiliations:** Florida Medical Entomology Laboratory, Institute of Food and Agricultural Sciences, University of Florida, 200 9th St. SE, Vero Beach, FL 32962 USA

**Keywords:** *Culicoides insignis*, Biting midges, Oviposition, Laboratory rearing, Colonization, Bluetongue virus, Epizootic hemorrhagic disease virus

## Abstract

**Background:**

*Culicoides insignis* is a confirmed vector of bluetongue virus (BTV) throughout the American tropics and a possible vector of epizootic hemorrhagic disease virus (EHDV) in Florida. Despite its importance, fundamental information on the biology and ecology of this vector species is lacking. In this study, we examined the oviposition of *C. insignis* under laboratory conditions, monitored the development of immature stages and attempted colonization of this species.

**Methods:**

Live *C. insignis* females were collected from the field using CDC-UV-LED traps, allowed to blood-feed on live chicken and given various natural substrates for oviposition in two-choice assays. The eggs deposited were transferred to 0.3% agar slants, and the hatched larvae were provided a diet of *Panagrellus redivivus* Linnaeus nematodes and the development of all immature stages was monitored.

**Results:**

*Culicoides insignis* females exhibited an overall oviposition preference for dishes containing mud from their larval habitat as gravid females deposited a significantly higher number of eggs on these dishes (35.3 eggs/female) than on controls (17.7 eggs/female). The ovipositing females also deposited a higher percentage of eggs on substrates with habitat mud and other organically enriched muds (≥ 75.2%) compared to controls (31.0%). The larvae developed successfully to adulthood on the nematode diet, exhibiting high overall larval survival rates (85.0%). Sex ratios of the F1 generation were male biased, approximately 3:1 (male:female). Captive mating could not be induced in the F1 adults.

**Conclusions:**

Mud from the larval habitat and other organically enriched muds provide strong oviposition cues to *C. insignis* under laboratory conditions. Further studies will be needed to identify the key biotic/abiotic factors influencing midge oviposition in the field. The agar/nematode method is effective for the rearing of *C. insignis* larvae. However, further studies will be needed to address the issue of male-biased sex ratios in the progeny and to examine the mating habits/cues of *C. insignis* in nature, which may provide clues towards inducing captive mating in the F1 adults.

**Graphical abstract:**

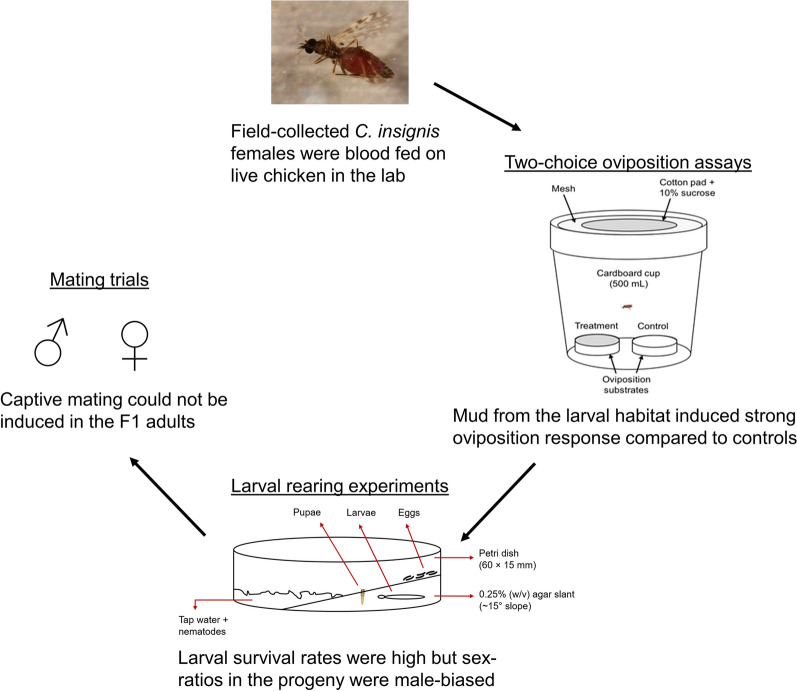

## Background

Orbiviruses such as bluetongue virus (BTV) and epizootic hemorrhagic disease virus (EHDV) affect ruminants and exert a significant negative impact on wildlife and animal farming industries worldwide. *Culicoides* species (Diptera: Ceratopogonidae) are the only known biological vectors of these viruses. These insects are not only important, but also critical for the transmission of these pathogens [1, 2]. Despite their importance, very little is known regarding the biology and ecology of *Culicoides* species associated with *Orbivirus* transmission to date. In the USA, the confirmed vectors of BTV/EHDV are *Culicoides sonorensis* and *Culicoides insignis* [3–5]. However, *Culicoides stellifer*, *Culicoides venustus*, *Culicoides debilipalpis* and other species of this genus may also be involved in virus transmission, particularly in the southeastern USA [6, 7]. In general, very few *Culicoides* species (*C. sonorensis* and *C. insignis*) have been fully incriminated in BTV/EHDV transmission (able to satisfy all four vector incrimination criteria established by the World Health Organization [WHO] [8]), mainly because vector incrimination studies on these insects are difficult to accomplish due to lack of viable colonies.

Much of our knowledge on the larval ecology of *Culicoides* vectors in North America comes from studies on *C. sonorensis*, a midge species best studied in the artificial wastewater ponds of California and also the only confirmed vector of BTV/EHDV successfully colonized to date worldwide [9–14]. As such, basic research on the biology, ecology and successful laboratory colonization of *C. insignis* and other *Culicoides* species associated with virus transmission in the southeastern USA would provide highly valuable information towards improving our understanding of the transmission dynamics of BTV/EHDV in this region. Furthermore, such information will also be useful in the development of effective vector management strategies in the long term. In this study, we examined the oviposition preferences and immature developmental traits of *C. insignis* under laboratory conditions and attempted colonization of this species. *Culicoides insignis* is a common vector of orbiviruses that is distributed across most of South America, Central America and the Caribbean, with its northern boundary extending into Florida and the neighboring states [15, 16]. This species is frequently associated with livestock facilities [17–19], is a significant biting pest of livestock [20, 21] and is a confirmed vector of BTV in Florida (all four vector incrimination criteria established by WHO satisfied) and also likely plays a role in EHDV transmission in the state [5, 22]. Overall, our study demonstrates effective methods for the blood-feeding, oviposition and egg collection of *C. insignis*, and provides the first insight into the oviposition preferences and immature developmental traits of this species, even though successful colonization was not achieved.

## Methods

### Live midge collection

Live midges were collected using procedures described previously for *C. stellifer* [23]. Briefly, CDC miniature light traps (model #2836BQ; BioQuip Products, Rancho Dominguez, CA, USA) fitted with UV-LED arrays (catalog #2790V390; BioQuip Products) and insect collection containers were set up overnight at the Archbold Biological Station’s Buck Island Ranch, Lake Placid, FL, USA (27°9′16.4″N, 81°11′51.7″W). The field-collected insects were brought to the laboratory the next morning where *C. insignis* females were anaesthetized using triethylamine (TEA) [23], morphologically identified [15], caged in paper cups (500 ml) and provided with 10% sucrose until being used for the oviposition studies.

### Oviposition studies

*Culicoides insignis* females were starved for 6–12 h after which they were introduced into 50-ml conical tubes fitted with fine mesh (in groups of 15–63) and placed on the breast of a live restrained chicken for about 45 min to blood-feed (University of Florida IACUC protocol #7682). The partially and fully engorged females were sorted under TEA anesthesia, placed individually in paper cups provided with two different substrates for oviposition (two-choice assays) and allowed to oviposit for 14 days.

Overall, four different two-choice assays were conducted in various combinations: (i) deionized water (DI) versus DI; (ii) DI versus habitat mud; (iii) habitat mud + cattle manure (25% w/w) versus habitat mud; (iv) habitat mud versus non-habitat mud. Each oviposition dish contained about 1 g of natural substrate placed on the bottom of a Petri dish (35 × 10 mm) that was covered with a layer of cotton and a filter paper on the top. The various natural sources tested for their oviposition attractiveness were selected based on personal observations and published reports [19, 24], with the objective of identifying factors serving as oviposition cues to gravid females. Emergence sampling was conducted on various sites across Florida to identify habitats and non-habitats of *C. insignis*. The larval habitat of *C. insignis* was identified on the property of Archbold Biological Station’s Buck Island Ranch, Lake Placid, FL, USA. Surface mud samples (top few centimeters) along the waterline at this site were collected into Ziploc bags using a trowel. Non-habitat mud was collected in the same way from a shaded puddle midge habitat located on a commercial cervid facility in Quincy, FL, USA [24]. Emergence sampling from this habitat indicated that this site was a productive habitat of *C. stellifer* and no *C. insignis* emerged from this site. Fresh cattle manure was collected from a cattle pasture in Vero Beach, FL, USA. During the oviposition bioassays, moisture levels across all dishes were maintained constant by adding 1.5 ml deionized water (DI) and replenishing with the same volume of DI in both dishes whenever required. The oviposition dishes were checked daily, and the number of eggs deposited on them were counted daily. After the end of the 2-week period or after midge death, females were dissected and the number of eggs retained, if any, were also counted. The females were provided with fresh cotton pads dampened with 10% sucrose solution daily. Each oviposition bioassay had five to eight replicates using a single midge in each replicate. At least two trials were conducted per each experiment (Table 1). All oviposition studies were conducted at 26 ± 1 °C and 60–80% relative humidity (RH) under a 14:10-h (light:day) photoperiod cycle.

**Table 1:** Summary of the oviposition experiments conducted on *Culicoides insignis*

Experiment	Two-choice oviposition preference assay	Trials (replicates, *n* )	Blood-fed females (*n*)	Gravid females, *n *(%)	Ovipositing females, *n* (%)	*P*-value
1	DI vs DI	2 (6)	12	10 (83.3)	4 (40.0)	0.3500
2	DI vs habitat mud	3 (6–8)	21	18 (85.7)	18 (100.0)	0.0780^a^
3	Habitat mud + cattle manure vs habitat mud	2 (6)	12	5 (41.7)	4 (80.0)	0.4200
4	Habitat mud vs non-habitat mud	2 (5–6)	11	8 (72.7)	8 (100.0)	0.3400
	Total females,* n*		56	41	34	

^a^Oviposition preference of *C. insignis* for mud substrates over DI substrates was marginally significant

### Larval rearing experiments

Midge larval rearing was conducted using methods described previously for *C. stellifer* [25]. Briefly, the eggs deposited during the oviposition studies (24–36 h old) were placed in Petri dishes (60 × 15 mm) containing 0.3% (w/v) agar slants and allowed to hatch. The larvae were fed a diet of *Panagrellus redivivus* Linnaeus nematodes (Carolina Biological Supply Company, Burlington, NC, USA) that were replenished every Monday, Wednesday and Friday (approx. 2 mg/day). The life history traits recorded were egg stage duration, egg hatch rates (of deposited eggs), larval survival rates to pupal stage, larval stage duration, pupal stage duration, adult eclosion rates and sex ratio of the emerged adults. Each Petri dish contained 10–20 eggs (from different females) with four to six dishes per trial. Three independent trials were conducted overall (Table 2). All larval rearing experiments were conducted at 26 ± 1 °C and 60–80% RH under a 14:10-h (light:day) photoperiod cycle.

**Table 2:** Summary of the larval rearing experiments conducted on *Culicoides insignis*

Trial (larval dishes,* n*)	Eggs/dish (*n*)	Egg stage duration (days)
1 (6)	20	3–4
2 (6)	10	3–4
3 (4)	10–11	3–4

### Statistical analysis

For the oviposition experiments, only gravid females were included in the statistical analyses. Variation in the number of eggs produced by gravid females and percentage of eggs retained by ovipositing females across the experiments were analyzed using generalized linear mixed-effects models (GLMM; package *lme4*) with the variation arising from trials and females incorporated as a random-effect under binomial or Poisson distributions. The oviposition preferences exhibited by *C. insignis* in the two-choice assays were analyzed using generalized estimating equations (GEE; package *geepack*) with a logit link function under binomial distribution taking into consideration the percentage of eggs retained by ovipositing females. As each midge was given two choices for oviposition, these dishes were regarded as a cluster and an exchangeable correlation structure was incorporated in the models [26]. Variation in the percentage of egg batch deposited (control + treatment) by females during different experiments was analyzed using generalized linear models (GLM) with a binomial distribution. For the larval rearing experiments, differences in the egg hatch rates, larval survival rates to pupal stage, larval stage duration, pupal stage duration, eclosion rate and F1 adult sex ratio between trials were analyzed using GLMM with the variation arising from females as a random effect under binomial or Poisson distributions. All data were analyzed using R statistical software v.3.6.1 using the packages *MASS*, *car*, *lme4* and *geepack* [27–31].

## Results

**Fig. 1:**
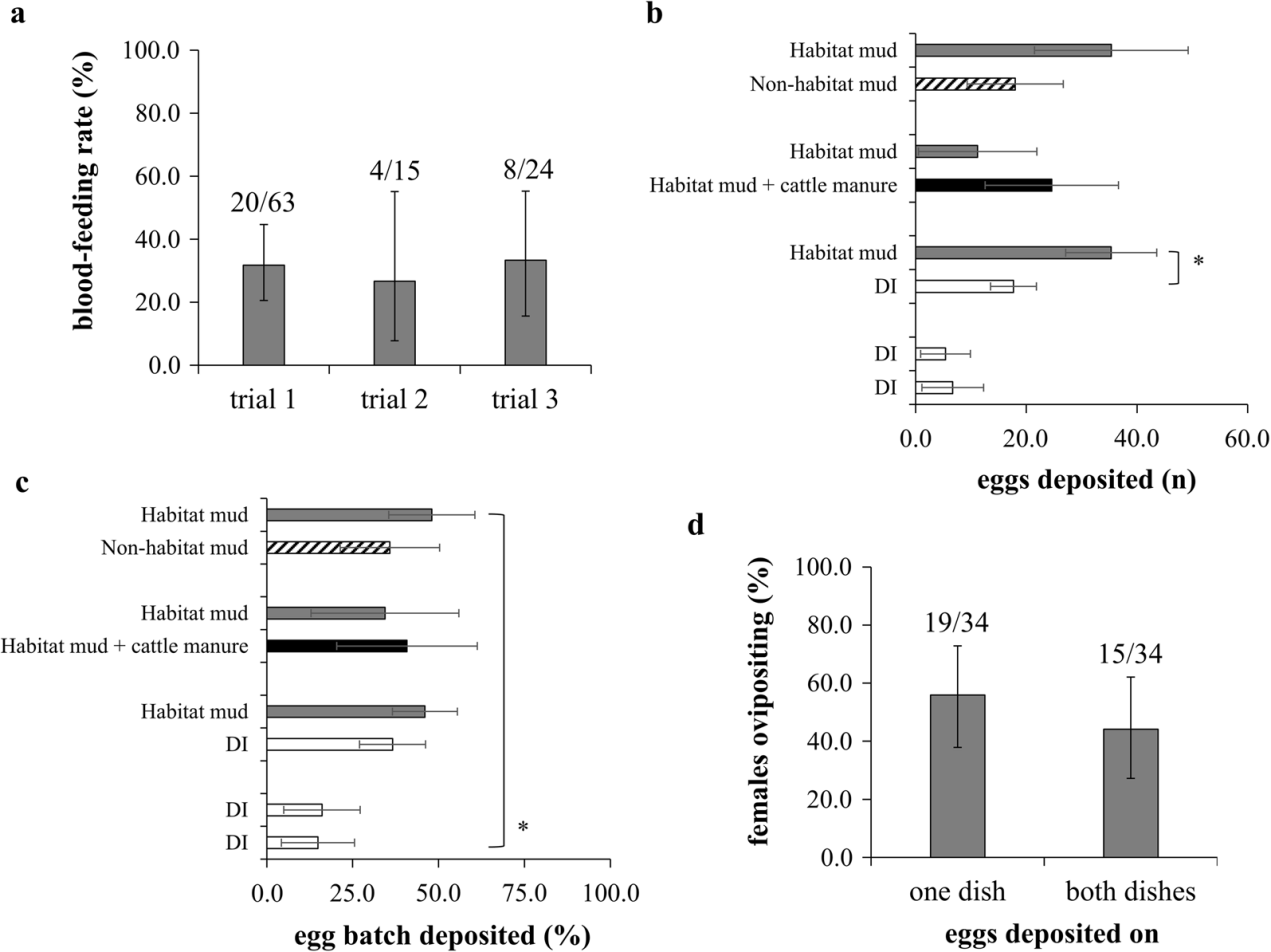
Life history traits of *Culicoides insignis* adult females under laboratory conditions. **a** Blood-feeding rates on live chicken (± 95% CI). Numbers above bars indicate the number of blood-fed midges/total number of midges. **b** Number of eggs deposited (mean ± SE). Asterisk indicates that a significantly higher (marginally; *P* = 0.0780) number of eggs were deposited on mud substrates compared to DI controls. **c** Percentage of egg batch deposited (mean ± SE). Asterisk indicates a significantly lower percentage of eggs were deposited during DI versus DI trials (on both dishes combined) than during other experiment trials). **d** Percentage of females that deposited eggs on one or both dishes (± 95% CI). Numbers above bars indicate the number of females depositing eggs on one or both dishes/total number of females that oviposited. Abbreviations: CI, Confidence interval; DI, deionized water; SE, standard error

Blood-feeding rates on live chicken ranged from 26.7 to 33.6% (overall mean ± standard error [SE]): 30.6 ± 2.0%) (Fig. 1a). For the oviposition experiments, a total of 56 *C. insignis* females were blood-fed across the study, which resulted in 41 females that developed eggs and 34 females that deposited eggs overall (Table 1). The percentage of females that developed eggs varied between experiments from 41.7% (5/12) during mud + cattle manure versus mud trials to 85.7% (18/21) during DI versus mud trials (Table 1). Fewer gravid females oviposited. Very few gravid females deposited eggs during DI versus DI trials (40.0%, 4/10) compared to other three experiment trials (≥ 80.0%) (Table 1). The number of eggs produced by gravid females ranged from 2 to 111 (mean ± SE: 56.1 ± 4.0 eggs/female) and varied significantly across the study (*χ*^2^ = 12.0, *df* = 3, *P* = 0.0074). During the double control trials (DI vs DI), very few eggs were deposited overall, with the number of eggs deposited on one dish (6.7 ± 5.6) not being significantly different from that deposited on the other (5.4 ± 4.5) (*χ*^2^ = 0.9, *df* = 1, *P* = 0.3500) (Fig. 1b). During the DI versus habitat mud trials, the number of eggs deposited on the substrates with habitat mud (35.3 ± 8.2) was significantly higher (marginally) than that deposited on DI substrates (17.7 ± 4.1) (*χ*^2^ = 3.1, *df* = 1, *P* = 0.0780). During the habitat mud + cattle manure versus habitat mud trials, the number of eggs deposited on habitat mud + cattle manure substrates (24.6 ± 12.0) was higher than that deposited on habitat mud substrates (11.2 ± 10.7) but the difference was not statistically significant (*χ*^2^ = 0.7, *df* = 1, *P* = 0.4200). Similarly, during the habitat mud versus non-habitat mud trials, the number of eggs deposited on habitat mud substrates (35.4 ± 13.9) was higher than that deposited on non-habitat mud substrates (18.0 ± 8.7), but the difference was not statistically significant (*χ*^2^ = 0.9, *df* = 1, *P* = 0.3400) (Fig. 1b). The percentage of egg batch deposited by females (control + treatment) was the lowest during the DI versus DI trials (31.0%) than during the other three experiment trials (≥ 75.2%) (*χ*^2^ = 627.0, *df* = 3, *P* < 0.0001) (Fig. 1c). Among the females that oviposited, 55.9% (19/34) deposited eggs on one dish, and the remaining females (44.1%, 15/34) deposited eggs on both dishes (Fig. 1d).

**Fig. 2:**
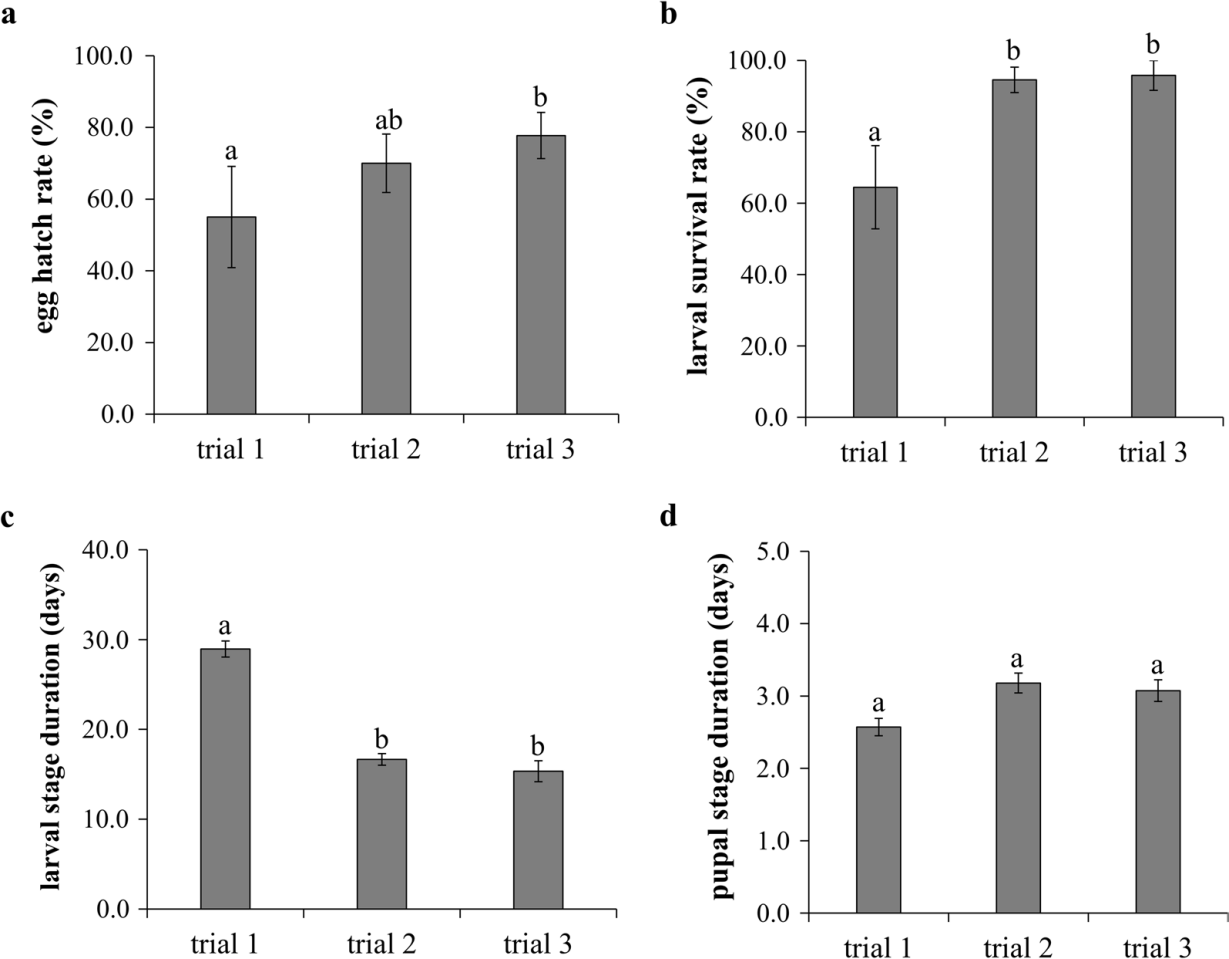
Life history traits of the immature stages of *C. insignis* under laboratory conditions. **a** Egg hatch rates (mean ± SE). **b** Larval survival rates. **c** Larval stage duration. **d** Pupal stage duration. Different lowcase letters above bars indicate significant differences between trials (*P* < 0.05)

**Fig. 3:**
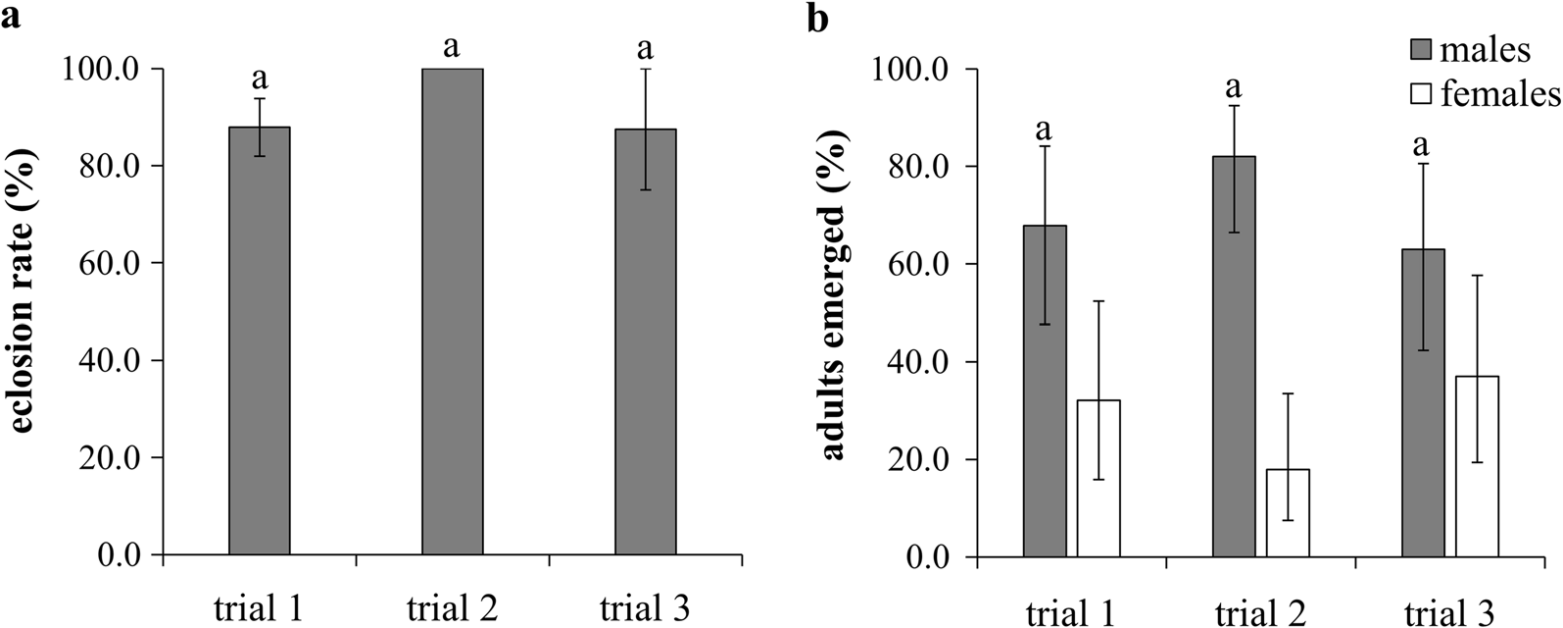
Life history traits of *C. insignis* observed in the study. **a** Eclosion rates (mean ± SE). **b** Sex ratios (± 95% CI). Different lowcase letters above bars indicate significant differences between trials (*P* < 0.05)

The egg stages of *C. insignis* lasted between 3 and 4 days (Table 2). The egg hatch rates ranged from 55.0 to 77.7% and differed significantly between the trials (*χ*^2^ = 8.3, *df* = 2, *P* = 0.0157) (Fig. 2a). Larval survival rates to the pupal stage ranged from 64.5 to 95.8% (overall mean ± SE: 85.0 ± 10.3%) and differed significantly between the trials (*χ*^2^ = 24.3, *df* = 2, *P* < 0.0001) (Fig. 2b). Larval stage duration ranged from 15.4 to 29.0 days (20.3 ± 4.3 days) and differed significantly between trials (*χ*^2^ = 193.1, *df* = 2, *P* < 0.0001) (Fig. 2c). Pupal stage duration ranged from 2.6 to 3.2 days (2.9 ± 0.2 days) and did not vary significantly between the trials (*χ*^2^ = 2.2, *df* = 2, *P* = 0.3385) (Fig. 2d). Adult eclosion rates from the pupal stage were high (≥ 87.5%) and showed no significant differences between the trials (*χ*^2^ = 0.1, *df* = 2, *P* = 0.9648) (Fig. 3a). Sex ratios of the F1 adults were male biased (approx. 3:1 [male:female]) and were not significantly different between trials (*χ*^2^ = 3.2, *df* = 2, *P* = 0.2016) (Fig. 3b).

## Discussion

Overall, our study demonstrates useful methods to collect live *C. insignis* midges from the field, blood-feed them under laboratory conditions, collect eggs from gravid females and rear the larvae to adulthood. In addition, our findings provide valuable insight into the life history traits of *C. insignis*, an important vector of BTV/EHDV in Florida. Although considered a mammal biter [32], *C. insignis* females showed satisfactory blood-feeding rates on live chicken in the laboratory. These blood-feeding rates can be possibly increased further by altering starvation periods or environmental conditions during blood-feeding or by using other laboratory animals (mammals) as a blood source. Moreover, the fecundity of *C. insignis* can also be potentially increased by using a mammalian blood source (vs. the avian blood source used in this study) as host blood meal source can alter fecundity in hematophagous species [33, 34]. However, further studies will be needed to test these hypotheses.

During the oviposition experiments, gravid females deposited a distinctly higher number of eggs on substrates with habitat mud than on the DI controls, suggesting that mud from the larval habitat provides strong oviposition cues to *C. insignis* under laboratory conditions. However, the number of eggs deposited on substrates during habitat mud + cattle manure versus habitat mud trials and habitat mud versus non-habitat mud trials were not significantly different, suggesting that organically enriched muds other than the habitat mud are also attractive for the oviposition of this species. It should be noted that these results should be interpreted cautiously as they could be an artifact of the small sample size of the study (only 12 females oviposited in these 2 experiments; Table 1). Furthermore, cattle had open access to this site on the Archbold ranch, and it is currently unknown whether the habitat mud of the *C. insignis* examined was already enriched with animal manure. Moreover, if olfactory cues are involved in the oviposition site selection of *C. insignis*, the set-up of the experimental design could have caused errors in the recognition of preferred substrates as the two dishes were placed close to each other in the relatively small sized paper cups (500 ml). Further studies using Y-shaped olfactometer bioassays could examine whether these results are biologically significant and determine whether these oviposition cues are olfactory/tactile in nature.

It currently remains unknown whether other natural sources from the habitat, such as vegetation, play a role in the oviposition of *C. insignis* (vegetated water bodies often harbor *C. insignis* larvae) [15, 19]. Previous studies found that mud and/or vegetation (*Sphagnum* spp. moss) from the larval habitat strongly influenced the oviposition of *C. stellifer* and *Culicoides impunctatus* under laboratory conditions [23, 35]. Currently, very little is known regarding the oviposition preferences and/or habitat requirements of *C. insignis* and other important *Culicoides* species in North America [15, 19, 23, 24]. Future studies characterizing the larval habitat of *C. insignis*, examining the physicochemical properties of the breeding site and identifying the key biotic/abiotic factors influencing oviposition site selection of this species in nature are warranted. This information, in the long term, can be potentially exploited to design novel sampling/control strategies targeting gravid females and to manipulate local habitats to discourage the oviposition of *C. insignis*. However, it is to be emphasized that the conclusions drawn from this study are based on experiments conducted under laboratory conditions. Therefore, any extrapolations to field conditions should be made cautiously.

The large variation in the number of eggs produced by blood-fed females in the study was not unexpected. It is likely that this variation was due to differences in the size of the blood meal ingested by females (larger blood meal sizes lead to higher number of eggs produced, and partially-engorged females were also included in the study) [36]. The huge variation in the percentage of females that developed eggs in the study was also not unexpected. It is likely that this was due to variation in the mated status of the females used in the study (some of the midges collected from the field may have been unmated and therefore could not produce viable eggs post blood meal) [37]. However, variation in the percentage of females that oviposited and variation in the percentage of egg batch deposited by ovipositing females likely represent a differential preference for the available oviposition substrates. For example, the few females that did oviposit during the study deposited a very small percentage of their egg batch during the DI versus DI trials compared to the other experiment trials, suggesting an avoidance of DI substrates and a preference for habitat mud and other organically enriched muds. Future studies that require oviposition and/or collection of eggs from *C. insignis* in the laboratory may benefit by providing organically enriched substrates to gravid females.

It was interesting that among the 34 females that oviposited across the study, 44% (15/34) demonstrated skip oviposition as they deposited eggs on both of the dishes available. Such behavior was documented previously in *C. stellifer* as well but appears to be more common in *C. insignis* than in *C. stellifer* (9% females) [23]. Skip oviposition has been well studied in numerous container-breeding mosquito species [23, 38–40]. It is believed that skip oviposition is advantageous in resource-limited habitats (such as artificial containers, plant pitchers, tree holes or others) as it enhances larval survival by reducing larval densities. However, the role of skip oviposition on the survival of mud-breeding species, such as *C. insignis* and *C. stellifer*, is currently unknown. Further studies will be needed to understand the role of skip oviposition on the ecology, survival and other life history traits of mud-breeding *Culicoides* species. Further studies will also be needed to examine whether or to what extent skip oviposition occurs in dung-breeding and tree-hole dwelling *Culicoides* species.

The egg hatch rates of *C. insignis* varied significantly across the study, which was not unexpected. It is likely that fertilization status of the eggs varied, possibly due to variation in the age of the field-collected females and/or the mated status of the males these females mated with in the field. The prior-mated status of males has been found to affect egg fertilization rates in tephritid flies and butterflies [41, 42]. The significant variation in larval survival rates and larval stage durations across the study were also not unexpected. It is possible that this variation arose due to: (i) variation in the age/nutritional status of females from which the eggs were obtained as parental nutrition can affect larval development traits in insects [43, 44]; (ii) variation in the number of eggs placed in the larval dishes (range: 10–20) as larval densities can affect insect larval development [45–47]; and/or (iii) age/condition of the nematodes used as midge larval diet as early-instar midge larvae may have had difficulties capturing/ingesting adult nematodes.

Overall, the agar/nematode method was convenient and effective for the larval rearing of *C. insignis*. All larval instars could be seen moving through the agar and in/out of the standing water freely. The late instars were frequently observed engulfing nematodes whole while the early instars probably fed on nematode pieces/carcasses and/or the microbial community of the medium. Interestingly, *C. insignis* larvae (late instars) were also observed to feed on dead conspecific larvae (on rare occasions), suggesting that *Culicoides* larvae are omnivorous opportunistic feeders. Pupation occurred mainly on the surface of the agar, but the pupae were also found floating in the standing water, albeit with at a lower frequency. Although the larval development of *C. insignis* was successful, the sex ratio of the F1 adults was male-biased, which may not be desirable for potential colony maintenance. The reasons behind this outcome are currently unknown. However, it is likely that the nematode diet used could not satisfy nutritional requirements of the female larvae potentially causing mortality. Previously, female mosquitoes were suggested to require more larval nutrition than males to pupate [48]. Moreover, previous larval rearing studies on *Culicoides* species using the agar/nematode method reported non-distorted sex ratios in the progeny only for *C. stellifer* and *C. circumscriptus* while the sex-ratios of other species were found to be either male-biased or female-biased [25, 49, 50]. It is likely that the larval nutritional requirements of *Culicoides* midges vary between species. Further studies will be needed to examine the nutritional requirements of male and female biting midge larvae, and to improve production conditions of *C. insignis* by potentially incorporating nutritional supplements to the nematode diet or by using other larval diets.

Very little is currently known about the mating behavior of *Culicoides* species. Many species are believed to be eurygamous (need swarming to mate) while some species are stenogamous (will mate in restricted spaces) [51–53]. Our attempts at inducing swarming/mating in the F1 generation of *C. insignis* by using host cues (octenol), environmental cues (habitat [mud + cattle manure] and dawn/dusk conditions), varying light colors (blue, green and red) and cage sizes (capillary tubes with terminalia in contact to large 47.5 × 47.5 × 47.5-cm BugDorm cages) were all unsuccessful (non-mating was inferred as F1 females did not deposit viable eggs post blood meal). The reproductive behavior of *C. insignis* has not been reported to date. Further studies will be needed to investigate the mating habits/cues of *C. insignis* in nature, which may offer clues towards providing conditions that encourage captive mating in this species.

## Data Availability

All data collected during this study have been statistically analyzed and published in this manuscript.

## Abbreviations

BTV: Bluetongue virus
DI: Deionized water
EHDV: Epizootic hemorrhagic disease virus
RH: Relative humidity
